# The genus *Nipponodrasterius* Kishii (Coleoptera, Elateridae, Agrypninae), a junior synonym of the genus *Gamepenthes* Fleutiaux (Coleoptera, Elateridae, Elaterinae), with review of the Japanese *Gamepenthes* species

**DOI:** 10.3897/zookeys.1004.56201

**Published:** 2020-12-16

**Authors:** Kôichi Arimoto, Hisayuki Arimoto

**Affiliations:** 1 Laboratory of Animal Ecology, Graduate School of Science, Kyoto University, Kyoto, 606–8502 Japan Kyoto University Kyoto Japan; 2 Tedukayama-nishi, Osaka, 558–0052 Japan Tedukayama-nishi Osaka Japan

**Keywords:** Japan, Megapenthini, new synonym, Oophorini, Palearctic region, taxonomic review

## Abstract

The genus *Nipponodrasterius* Kishii, 1966 was established as a member of the subfamily Conoderinae Fleutiaux, 1919 (now tribe Oophorini Gistel, 1848; subfamily Agrypninae Candèze, 1857) based on *N. alpicola*. The genus was suggested to be unlikely to belong to Agrypninae because it lacks diagnostic features of the Agrypninae. However, there are no taxonomic treatments for the genus or species. Here, we review the status of the genus and species by examining the holotype of *N. alpicola*. Consequently, the genus was found to be a junior synonym of the genus *Gamepenthes* Fleutiaux, 1928 and *N. alpicola* was found to be a junior synonym of *G. pictipennis* (Lewis, 1894). We review all species of *Gamepenthes* in Japan and provide a key to species.

## Introduction

The monotypic elaterid genus *Nipponodrasterius* Kishii, 1966 was established as a member of the subfamily Conoderinae Fleutiaux, 1919 (currently tribe Oophorini Gistel, 1848; subfamily Agrypninae Candèze, 1857) based on *Nipponodrasterius alpicola* Kishii, 1966. This species is known only from the holotype collected on a mountain (altitude 2800 meters) in central Honshu, Japan. Its sex was not determined in the original description. The original description separates the genus from known members of tribe Oophorini using the following characteristics: rather flat body, antennomeres II and III small, antennomeres IV–X clearly serrate, elytral surface clearly granulated, and tarsomere IV shortly bilobed. Kishii (1987) redescribed this genus, modifying the original description slightly. He changed the description “tarsomere IV shortly bilobed” in Kishii (1966) to “tarsomere IV slightly enlarged apically”. Moreover, he added an important character state to the generic description, claws without basal setae. Ôhira (1994) focused on the status of the claws and suggested that this genus is unlikely to belong to Agrypninae, because the presence of basal setae on the claws is a diagnostic feature of members of Agrypninae, absent only in genus *Danosoma* Thomson, 1859 (Hayek 1973). Ôhira (1994) did not examine specimens of the genus directly and did not revise the status of the genus.

We observed that *N. alpicola* is similar to species of the genus *Gamepenthes* Fleutiaux, 1928 in the tribe Megapenthini Gurjeva, 1973, subfamily Elaterinae Leach, 1815 based on these diagnostic features and a drawing of the dorsal habitus in the original description. In considering the systematic position of *N. alpicola*, the direction of the mouth-parts and the structure of the head capsule, procoxal cavity, prosternal process, mesocoxal cavity and mesosternum are also important characters (Ôhira 1970a; Stibick 1979; Schimmel 2003), however, these features were not described in Kishii (1966, 1987). The holotype of the type species required re-examination.

Five *Gamepenthes* species are distributed in Japan: *G. ornatus* (Lewis, 1894); *G. pictipennis* (Lewis, 1894); *G. similis* (Lewis, 1894); *G. versipellis* (Lewis, 1894); *G. yoshidai* Ôhira, 1995. Ôhira (1995a) reviewed the four Japanese species, except for *G. yoshidai*, and showed that they are clearly separated from each other by their aedeagi. Ôhira (1995a) provided a key to distinguish them, based mainly on body color, although members of the genus often show color variation. Subsequently, Ôhira (1995b) described a new species as *G. yoshidai* without showing its aedeagus. Hence, we examine the holotype of *N. alpicola* and review the Japanese *Gamepenthes* species including *G. yoshidai* in order to resolve the problem of *Nipponodrasterius*. Additionally, we provide a new key to these species based on external morphology.

## Material and methods

We examined the holotype of *N. alpicola* and non-type specimens of the five Japanese *Gamepenthes* species, as well as the only paratype of *G. yoshidai*. The type specimens are in the collection of the Osaka Museum of Natural History (OMNH; Osaka, Japan). The non-type specimens are in the personal collection of the authors (Osaka, Japan) and will be donated to the OMNH in the future. Unique identifier numbers of the non-type specimens are: GO001–GO014; GP001–GP012; GS001–GS010; GV001–GV017; GY001, GY002. We found a slide of the male genitalia of the holotype of *N. alpicola* in OMNH (Fig. 5L), although its sex and genitalia were not mentioned in the original description. On the slide, the aedeagus of the holotype, missing the phallobase, had been mounted in balsam and distorted due to pressure exerted by the cover slip (Fig. 5J).

The methods used for observing and dissecting specimens, taking photographs, creating line drawings, and depositing the dissected parts follow Arimoto and Suzuki (2020).

The classification of the family Elateridae follows Kundrata et al. (2019), Bouchard et al. (2011), and Cate (2007). The morphological nomenclature system follows Costa et al. (2010) and Douglas (2011). The diagnosis of *Gamepenthes* was based on information from Ôhira (1970a, 1995a), Schimmel (2003), and evidence found during this study. The non-type specimens were identified using descriptions and figures in Lewis (1894) and Ôhira (1995a, b).

### Measurements and indices

Measurements and indices were made following Arimoto and Suzuki (2020). Measurements are shown in millimeters. The following abbreviations are used:

**BL** Body length from head to elytral apices

**BW** Maximum body width

**MAE** Maximum distance across the eyes

**MBE** Minimum distance between the eyes

**OI** Ocular index: MAE/MBE × 100

**PL** Maximum pronotum length including hind angles

**PML** Length of the midline of pronotum

**PAW** Minimum pronotum width between anterior angles

**PW** Maximum pronotum width including hind angles

**PI** Pronotum index: PL/PW × 100

**PWI** Pronotum width index: PAW/PW × 100

**EL** Maximum elytra length

**EW** Maximum elytra width

**EI** Elytra index: EL/EW × 100

**BI** Body index: EL/PL × 100

## Taxonomy

### Genus Gamepenthes

Taxon classificationAnimalia ColeopteraElateridae

Fleutiaux, 1928

9BF51533-83B9-55A0-B22F-C433DEAE64E8

Gamepenthes Fleutiaux, 1928: 158 (original description; type species: Megapenthes octomaculatus Schwarz, 1898; by original designation).
Nipponodrasterius Kishii, 1966: 9 (original description; type species: Nipponodrasterius alpicola Kishii, 1966; by original designation); Kishii 1987: 62 (redescription). **syn. nov.**

#### Diagnosis.

Head capsule oval in lateral view. Supra-antennal carina complete, rounded, depressed medially. Frontoclypeal region narrowed medially. Mouth-parts inferior. Antennae serrate from antennomere IV, without median longitudinal carina; antennomere IV longer than II–III combined in many, shorter than II–III combined in a few. Pronotum with median basal furrow, without sublateral incision near hind angles; hind angles unicarinate. Pronotosternal sutures not grooved or very shallowly grooved in front. Posterior edge of hypomeron straight mesally and then broadly rounded. Procoxal cavity partly closed behind by mesal projection of hypomeron. Prosternal process concave between procoxae, with subapical tooth in lateral view. Side of scutellum parallel on anterior half. Mesocoxal cavity open to mesepimeron and mesepisternum. Mesosternum separated by suture from metasternum. Outer edge of metacoxal plates wide in most, but narrowed in a few. Elytral surface with rasp-like punctures; elytral apical edge serrate and with small spines, serration very slight in some and then almost rounded. Tarsi simple; claws simple, without basal setae.

Ôhira (1970a, 1995a) stated that the apical elytral edge is more or less truncate; however, this is based on a misunderstanding.

#### Distribution.

Oriental region (Bhutan, China, India, Indonesia, Laos, Malaysia, Myanmar, Nepal, Philippines, Taiwan, Thailand, Vietnam): 30 species (Schimmel 2003, 2004, 2006; Schimmel and Tarnawski 2009). Palearctic region (Japan, Russia): five species (Kishii 1999; Prosvirov 2013).

#### Ecology.

Adults of the genus are often observed visiting flowers during the daytime. In Japan, *Gamepenthes* has been recorded visiting the flowers of *Sambucus* sp. and *Viburnum* sp. (family Adoxaceae), *Angelica* sp. (Apiaceae), *Aralia cordata* Thunb. (Araliaceae), *Clethra barbinervis* Siebold et Zucc. (Clethraceae), *Neoshirakia japaonica* (Siebold et Zucc.) Esser (Euphorbiaceae), *Castanea crenata* Siebold et Zucc. (Fagaceae), *Hydrangea paniculata* Siebold and H. serrata (Thunb.) Ser. var. acuminata (Siebold et Zucc.) Nakai (Hydrangeaceae), and *Cimicifuga* sp. (Ranunculaceae) (Ôhira, 1995a). This study adds flowers of *Tilia japonica* (Miq.) Simonk. (Malvaceae) to the records.

While most adult *Gamepenthes* individuals are not attracted to lights at night, *G. similis* has been collected by light traps (Ootsuka et al., 1981). We examined a few specimens of *G. ornatus* and *G. pictipennis* collected using simple light traps made with weak fluorescent lights.

#### Included species from Japan.

Five species: *G. ornatus* (Lewis, 1894); *G. pictipennis* (Lewis, 1894); *G. similis* (Lewis, 1894); *G. versipellis* (Lewis, 1894); *G. yoshidai* Ôhira, 1995.

### Key to species of the genus *Gamepenthes* from Japan

**Table:** 

1	Body size large (BL: over 5.1 mm) (Fig. 1A, B, D, E); antennomere II shorter than wide in male (Fig. 3A, C), almost as long as wide or slightly longer than wide in female (Fig. 3B, D); antennomere IV longer than II–III combined (Fig. 3A–D)	**2**
–	Body size small (BL: under 5.0 mm) (Fig. 1C); antennomere II longer than wide (Fig. 3E, F); antennomere IV shorter than II–III combined (Fig. 3E, F)	***G. similis* (Lewis, 1894)**
2	Antennomere IV robust (1.36–1.72 times longer than wide in male, 1.66–1.79 times longer than wide in female) (Fig. 3A, B); lateral half of metacoxal plates not narrowed laterad, with sides wide (Fig. 3G); apical edge of elytra serrate (Fig. 3I)	**3**
–	Antennomere IV elongate (1.74–2.06 times longer than wide in male, 1.96–2.17 times longer than wide in female) (Fig. 3C, D); lateral half of metacoxal plates narrowed laterad, with sides narrow (Fig. 3H); apical edge of elytra rounded, without spines (Fig. 3J)	**4**
3	Body size smaller (BL: under 6.5 mm in male, under 7.1 mm in female) (Fig. 1A); pronotum moderately widened posterad (PWI: 150–156 in male, 161–174 in female) (Fig. 4A, B)	***G. ornatus* (Lewis, 1894)**
–	Body size larger (BL: over 6.5 mm in male, over 7.0 mm in female) (Fig. 1D); pronotum strongly widened posterad (PWI: 172–184 in male, 176–191 in female) (Fig. 4C, D)	***G. versipellis* (Lewis, 1894)**
4	Body robust (EI: 222–244, BI: 209–247) (Fig. 1B); prosternal process weakly inclined dorsad (20–25°) (Fig. 4E)	***G. pictipennis* (Lewis, 1894)**
–	Body slender (EI: 259–265, BI: 254–282) (Fig. 1E); prosternal process strongly inclined dorsad (43–50°) (Fig. 4F)	***G. yoshidai* Ôhira, 1995**

**Figure 1.**
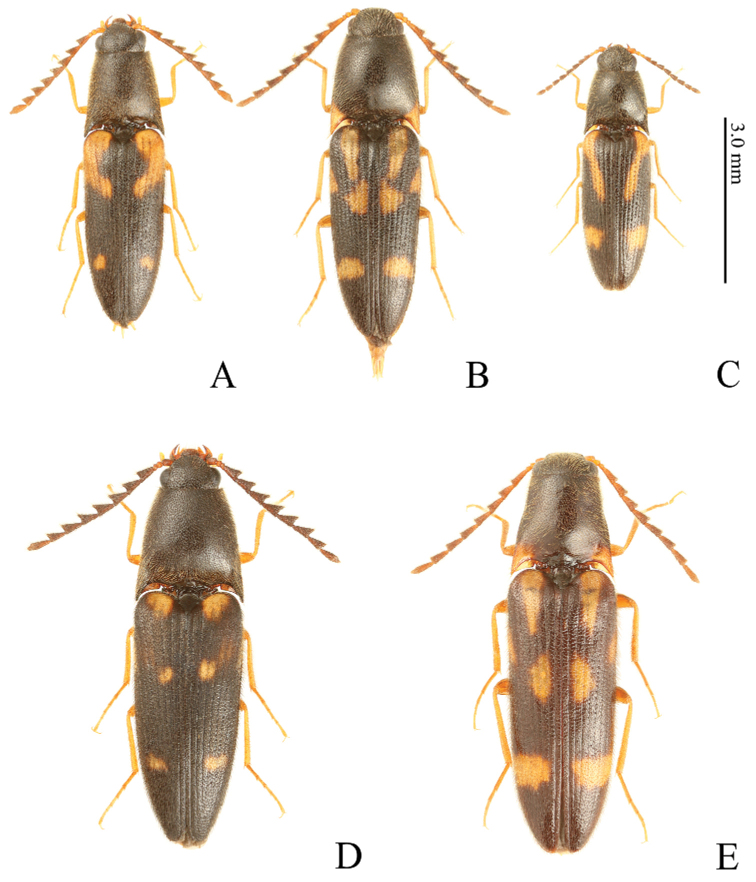
*Gamepenthes* spp., male, habitus, dorsal view **A***G. ornatus* [GO003] **B***G. pictipennis* [GP010] **C***G. similis* [GS003] **D***G. versipellis* [GV006] **E***G. yoshidai* [GY001].

**Figure 2.**
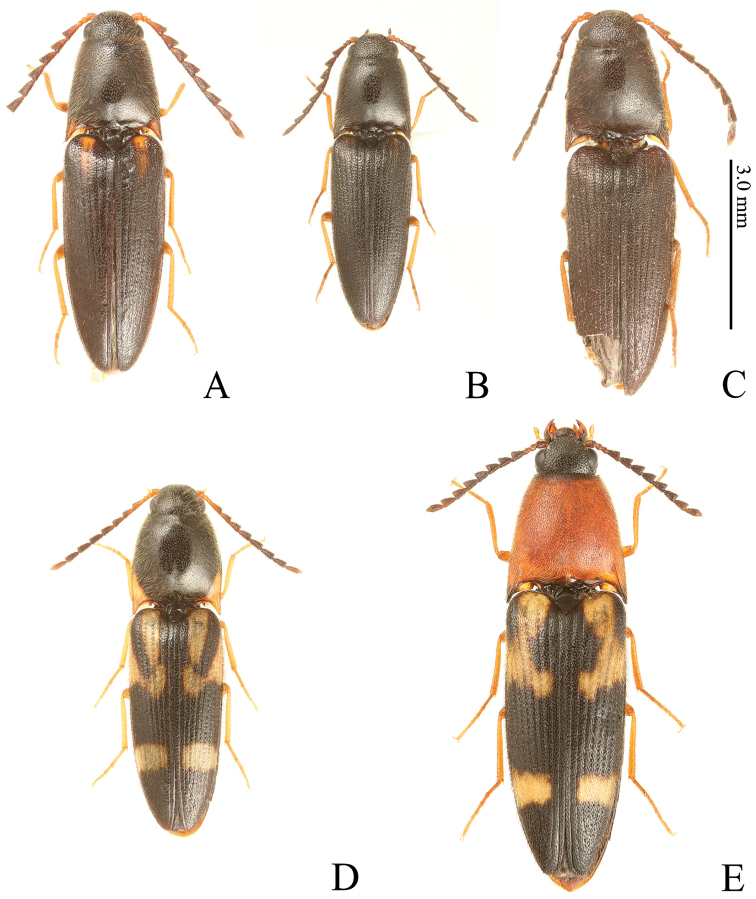
*Gamepenthes* spp., color variation **A***G. pictipennis*, male [GP003] **B***G. pictipennis*, male [GP012] **C***G. pictipennis*, male, holotype of *Nipponodrasterius alpicola***D***G. pictipennis*, female [GP010] **E***G. versipellis*, female [GV007].

### Gamepenthes ornatus

Taxon classificationAnimalia ColeopteraElateridae

(Lewis, 1894)

C8584265-4B22-5603-8458-3B00F6F2B96F

**Figure 3.**
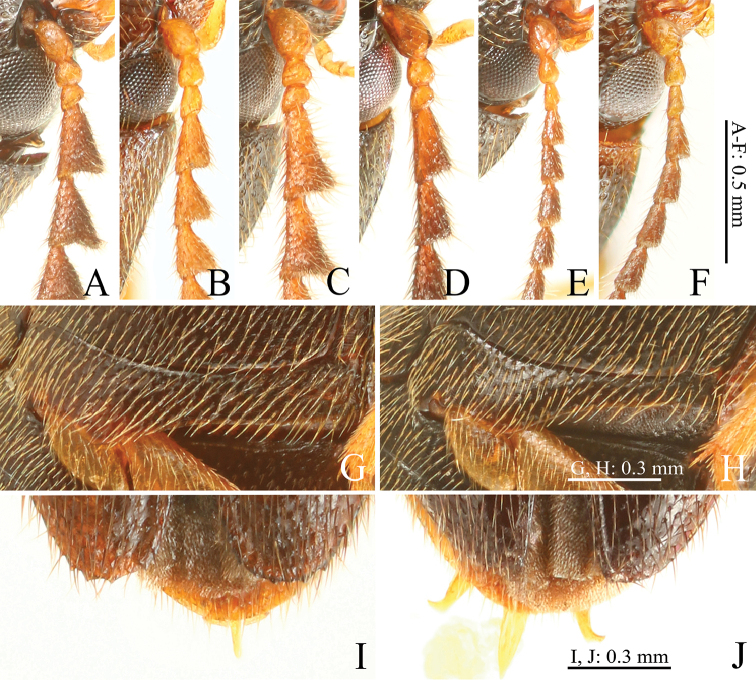
*Gamepenthes* spp. **A–F** right basal antennomeres **G, H** left metacoxal plate **I, J** apices of elytra **A***G. ornatus*, male [GO003] **B***G. ornatus*, female [GO009] **C***G. pictipennis*, male [GP010] **D***G. pictipennis*, female [GP011] **E***G. similis*, male [GS003] **F***G. similis*, female [GS006] **G, I***G. versipellis* [GV011] **H***G. pictipennis* [GP010] **J***G. pictipennis* [GP003].

**Figure 4.**
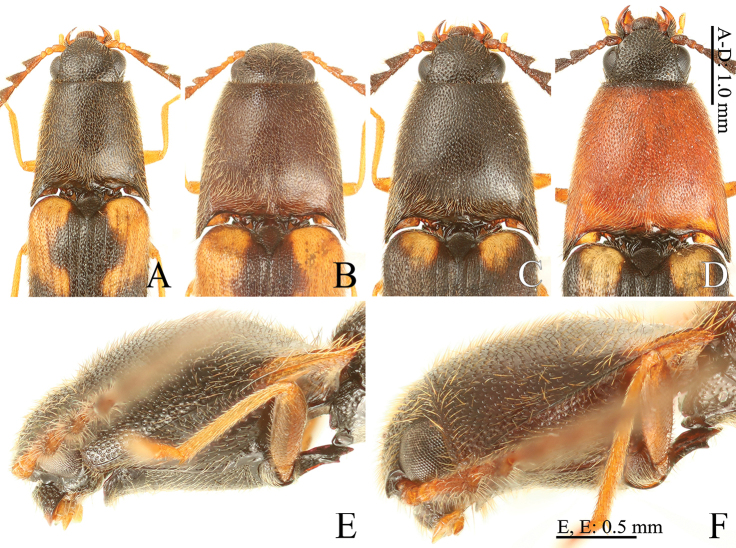
*Gamepenthes* spp., head and prothorax **A***G. ornatus*, male [GO003] **B***G. ornatus*, female [GO002] **C***G. versipellis*, male [GV006] **D***G. versipellis*, female [GV007] **E***G. pictipennis*, male [GP010] **F***G. yoshidai*, male [GY001] **A–D** dorsal view **E, F** lateral view.

Figures 1A , 3A, B , 4A, B , 7A

Megapenthes ornatus Lewis, 1894: 47 (original description; type locality: Yuyama, Kumamoto Prefecture, Kyushu, Japan).
Gamepenthes ornatus (Lewis, 1894); Kishii 1959: 58 (changed generic status).
Megapenthes ornatus aberrant form basalis Nakane, 1958: 87 (original description; type locality: Mt. Osore-zan, Aomori Prefecture, Honshu, Japan).

#### Material examined.

2 females, Japan, Honshu, Nara Prefecture, Yoshino District, Kamikitayama Village, Amagase, 11 VII 2004, Hisayuki Arimoto leg. [females: GO001, GO002]; 1 male, Japan, Honshu, Nara Prefecture, Yoshino District, Totsukawa Village, Asahi, Mt. Syakaga-take, 1450 m, 4 VIII 2019, Hisayuki Arimoto leg., from flowers of *Tilia japonica* [male:GO003]; 3 males, 1 female, Japan, Honshu, Wakayama Prefecture, Tanabe City, Mt. Gomadan-zan, 18 VII 2019, Hisayuki Arimoto leg. [males: GO004–GO006; female: GO007]; 2 females, Japan, Honshu, Nara Prefecture, Tenkawa Village, near Gyôjagaeshi Tunnel, 900 m, 16 VII 2017, Hisayuki Arimoto leg. [GO008, GO009]; 1 male, Japan, Honshu, Wakayama Prefecture, Tanabe City, Mt. Jyôgamori-yama, 18 VII 2019, Hisayuki Arimoto leg. [GO010]; 1 female, Japan, Honshu, Okayama Prefecture, Tomada District, Kagamino Town, Neji, 8–15 VII 2016, Akihiko Watanabe leg., by light FIT [GO011]; 1 male, Japan, Kyushu, Kumamoto Prefecture, Uki City, Mt. Shiraiwa-yama, 16–17 VII 2016, Ryô Noda leg. [GO012]; 2 females, Japan, Kyushu, Miyazaki Prefecture, Nishiusuki District, Gokase Town, Mt. Shiraiwa-yama, 1347 m, 32°34'13.7"N, 131°06'51.6"E, 30 VII 2011, Kôichi Arimoto leg. [GO013, GO014].

#### Diagnosis.

Body robust (EI: 238–254, BI: 220–236). Prothorax black in male, brown in female. Each elytron with pale orange U-shaped marking basally. Antennomere II shorter than wide in male, almost as long as wide in female; III shorter than wide in male, longer than wide in female; IV robust (1.36–1.53 times longer than wide in male, 1.69–1.79 times longer than wide in female), longer than II–III combined (1.53–1.99 times as long as II–III combined in male, 1.02–1.10 times as long as in female). Prosternal process weakly inclined dorsad (24–38°). Metacoxal plates not narrowed outwards on lateral half; outer edge wide. Apical edge of elytra serrate. Apex of parameres beyond pre-apical expansions equilateral triangular (apex length 0.8–0.9 times width of parameres at expansion).

#### Measurements.

Male (6 spec.). BL: 5.29–6.18; BW: 1.41–1.72; MAE: 0.893–1.07; MBE: 0.599–0.719; OI: 148–154; PL: 1.47–1.82; PML: 1.24–1.51; PAW: 0.925–1.08; PW: 1.41–1.70; PI: 105–107; PWI: 150–156; EL: 3.47–4.12; EW: 1.41–1.72; EI: 238–246; BI: 226–236. Female (8 spec.). BL: 5.86–7.03; BW: 1.57–1.93; MAE: 0.929–1.12; MBE: 0.620–0.764; OI: 141–150; PL: 1.71–2.12; PML: 1.42–1.81; PAW: 0.956–1.14; PW: 1.57–1.93; PI: 106–111; PWI: 161–174; EL: 3.90–4.67; EW: 1.57–1.93; EI: 242–254; BI: 220–230.

#### Distribution.

Japan: Honshu, Shikoku, Kyushu.

#### Comparative notes.

*Gamepenthes ornatus* and *G. versipellis* (Lewis, 1894) are often found sympatrically and are remarkably similar. They are distinguished by apical expansion of parameres beyond apical-lateral hooks (*G. ornatus*, apex length 0.8–0.9 times width of parameres at expansion; *G. versipellis*, 0.5–0.6 times width of parameres at expansion) (Fig. 7A, D).

Ôhira (1995a) distinguished *G. ornatus* from *G. versipellis* in the key by the antennomere II (*G. ornatus*, longer than wide; *G. versipellis*, shorter than wide) and color of the basal outer edge of the elytra (*G. ornatus*, pale orange, Fig. 1A; *G. versipellis*, black, Fig. 1D). However, in both species, the males’ antennomere II is shorter than wide (Fig. 3A), and in females, it is almost as long as or slightly longer than wide (Fig. 3B). Moreover, their elytral base is variable in color, and we found specimens [GO001, GO016] of *G. versipellis* with a pale orange basal outer edge of the elytra. The two species are difficult to distinguish using antennomere II and elytral color. In females, pronotum coloration is a good diagnostic character (*G. ornatus*, brown, Fig. 4B; *G. versipellis*, red, Fig. 4D) because no pronotum color variation has been found in either species. Considering the possibility that females with non-red pronota have been found, pronotum shape is the best diagnostic character for both sexes of the two species except for aedeagus (*G. ornatus*, PWI 150–156 in male and 161–174 in female; *G. versipellis*, PWI 172–184 in male and 176–191 in female) (Fig. 4A–D).

### Gamepenthes pictipennis

Taxon classificationAnimalia ColeopteraElateridae

(Lewis, 1894)

272DD5BF-63D4-5BC3-812A-31E9B1FA93C3

**Figure 5.**
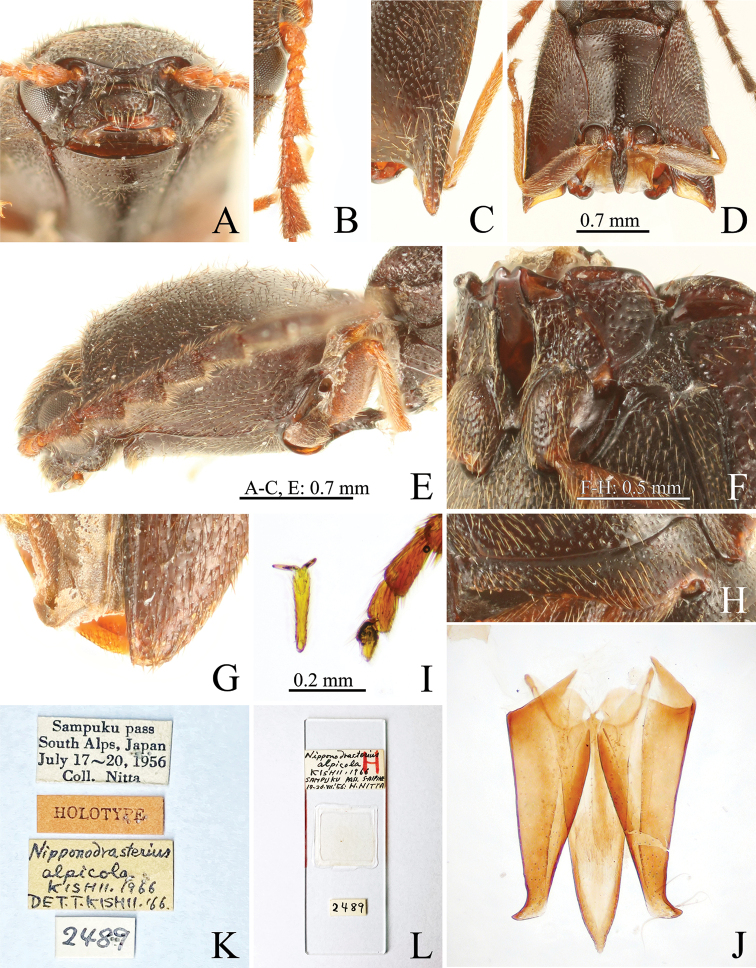
Holotype of *Nipponodrasterius alpicola***A** head, anterior view **B** right basal antennomeres **C** hind angles of pronotum **D** prothorax, ventral view **E** prothorax, lateral view **F** mesosternum **G** apices of elytra **H** right metacoxal plate **I** left mid tarsus and claw **J** aedeagus **K** labels **L** aedeagus slide.

**Figure 6.**
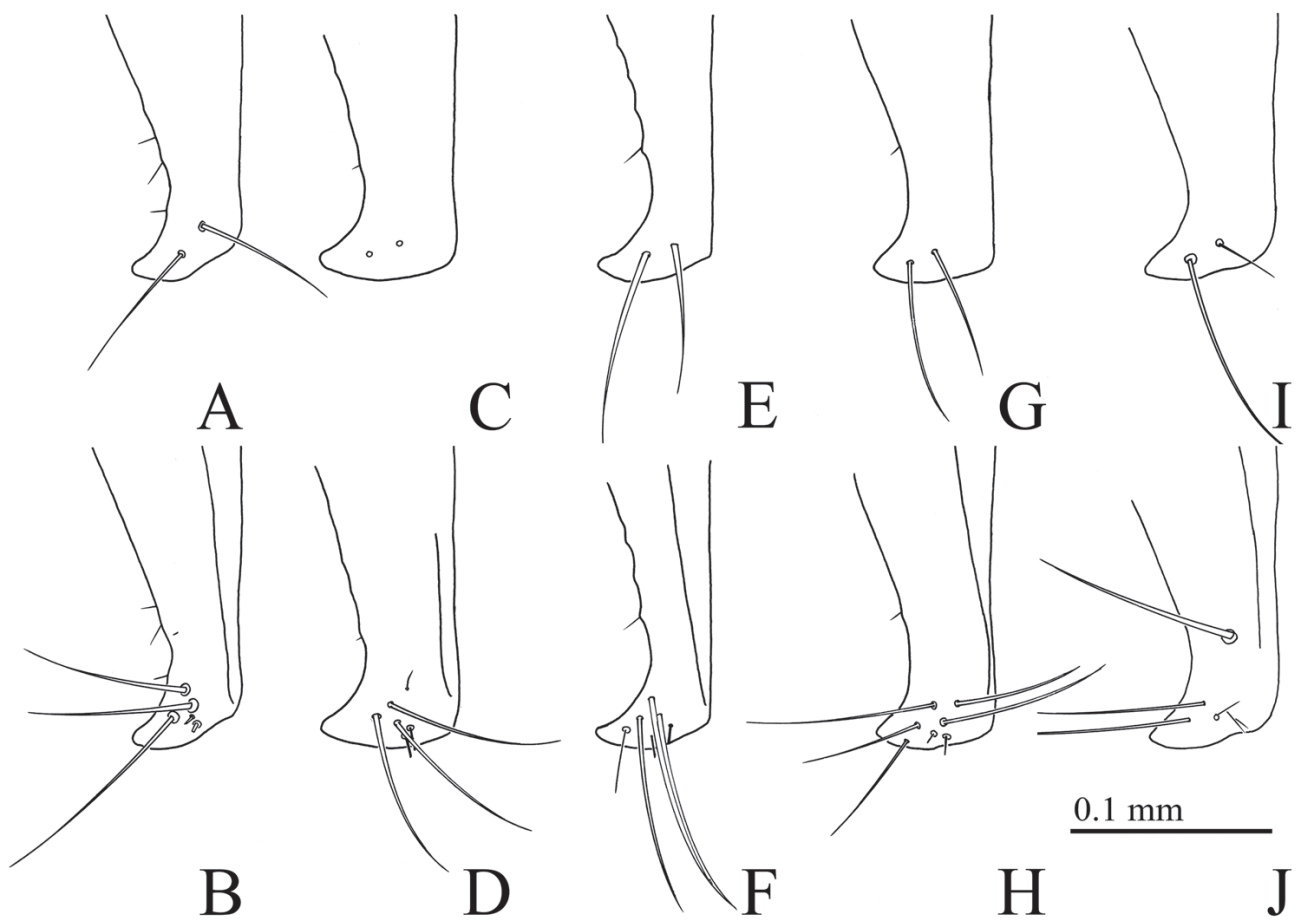
*Gamepenthes pictipennis*, male, apex of left paramere of aedeagus **A, B** [GP006] **C, D** [GP007] **E, F** [GP009] **G, H** [GP012] **I, J** holotype of *Nipponodrasterius alpicola***A, C, E, G, I** dorsal side **B, D, F, H, J** ventral side.

**Figure 7.**
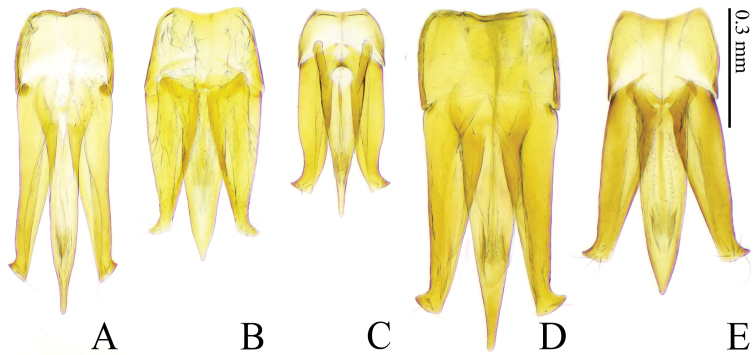
*Gamepenthes* spp., male, aedeagus, ventral side **A***G. ornatus* [GO006] **B***G. pictipennis* [GP006] **C***G. similis* [GS008] **D***G. versipellis* [GV003] **E***G. yoshidai* [GY002].

Figures 1B , 2A–D , 3C, D , 4E , 5 , 6 , 7B

Melanoxanthus pictipennis Lewis, 1894: 48 (original description; type locality: Fukushima [Kisofukushima in Nagano Prefecture] and Nataksugawa [Nakatsugawa in Gifu Prefecture], Honshu, Japan).
Gamepenthes pictipennis (Lewis, 1894); Kishii 1959: 58 (changed generic status).
Gamepenthes pictipennis aberrant form mizunoi Kishii, 1968: 14 (original description; type locality: Tokugô-tôge Pass, Nagano Prefecture, Honshu, Japan).
Nipponodrasterius alpicola Kishii, 1966: 9 (original description; type locality: Sampuku-tôge Pass, Kashio, Ôshika Village, Shimoina District, Nagano Prefecture, Honshu, Japan). **syn. nov.**

#### Material examined.

***Holotype*** of *Nipponodrasterius alpicola*. Male, Japan, Honshu, Nagano Prefecture, Shimoina District, Ôshika Village, Kashio, Sampuku-tôge Pass, 17–20, VII, 1956, H. Nitta leg. Verbatim label data (Fig. 5J): “*Nipponodrasterius* / *alpicola* / Kishii. 1966 / DET. T. KISHII. ‘66”; “HOLOTYPE”; “Sampuku pass / South Alps, Japan / July 17~20, 1956 / Coll. Nitta”; “2489”.

#### Non-type material.

1 male, Japan, Hokkaido, Uryû District, Horokanai Town, Syumarinai, 31 VII 1982, Koji Hosokawa leg. [GP001]; 1 female, Japan, Honshu, Gunma Prefecture, Ôsawa, 19 VII 1975, M. Yagi leg. [GP002]; 1 male, Japan, Honshu, Yamanashi Prefecture, Sudama Town, Masutomi spa, 9 VIII 1989, Yutaka Ishikawa leg. [GP003]; 1 female, same place as the former, 1300 m, 26 VII 1999, Yutaka Ishikawa leg. [GP004]; 1 female, same place as the former, ca 1300 m, base of the western end of the Okuchichibu Mountains, 18 VII 1990, Yutaka Ishikawa leg. [GP005]; 1 male, Japan, Honshu, Yamanashi Prefecture, Kôshû City, Enzan, Hikawa Forest Road, 20 VIII 1989, K. Shindô leg. [GP006]; 1 male, Japan, Honshu, Gifu Prefecture, Takayama City, Shôkawa Town, Oganigô, Ôkuro Valley, 30 VI 2007, Hisayuki Arimoto leg. [GP007]; 1 female, Japan, Honshu, Gifu Prefecture, Ôno District, Shôkawa Village, Ogamigô, 12 VIII 1995, N. Yuzawa leg. [GP008]; 1 male, Japan, Honshu, Nara Prefecture, Tenkawa Village, Tsubonouchi Forest Road, 1200m, 21 VII 2008, Hisayuki Arimoto leg. [GP009]; 1 male, 1 female, Japan, Honshu, Okayama Prefecture, Tomada District, Kagamino Town, Neji, 8–15 VII 2016, Akihiko Watanabe leg., by light FIT [male: GP0010; female: GP011]; 1 male, Japan, Kyushu, Ôita Prefecture, Toyogoôno City, Ogata Town, Mt. Furusobo-san, 30 VII 2017, Yûji Tsutsumiuchi leg. [GP012].

#### Diagnosis.

Body robust (EI: 222–244, BI: 209–247). Prothorax black; hind angles of pronotum yellow to orange in many, black in some. Each elytron with three separate yellow markings basally; basal markings connected in many, variably reduced in some. Antennomere II shorter than wide; antennomere III shorter than wide in male, slightly longer than wide in female; IV elongate (1.74–2.00 times longer than wide in male, 1.96–2.05 times longer than wide in female), longer than II–III combined (1.47–1.79 times as long as II–III combined in male, 1.21–1.40 times as long as II–III combined in female). Prosternal process weakly inclined dorsad (20–25°). Metacoxal plates narrowed outwards, with outer edge narrow. Apical edge of elytra rounded, without spines. Apex of parameres beyond pre-apical expansions widely triangular (apex length 0.1–0.2 times width of parameres at expansion).

#### Measurements.

Male (8 spec.). BL: 5.13–6.51; BW: 1.42–1.88; MAE: 0.903–1.10; MBE: 0.623–0.793; OI: 132–145; PL: 1.34–1.85; PML: 1.27–1.62; PAW: 0.937–1.13; PW: 1.37–1.76; PI: 101–111; PWI: 146–161; EL: 3.32–4.29; EW: 1.42–1.88; EI: 222–238; BI: 212–247. Female (5 spec.). BL: 5.65–7.25; BW: 1.55–1.96; MAE: 0.915–1.14; MBE: 0.653–0.856; OI: 133–140; PL: 1.61–2.11; PAW: 0.943–1.17; PML: 1.40–1.87; PW: 1.47–1.91; PI: 108–112; PWI: 155–163; EL: 3.57–4.72; EW: 1.55–1.96; EI: 228–244; BI: 209–237.

#### Distribution.

Japan: Hokkaido, Honshu, Shikoku, Kyushu. Russia: Kunashir Island. The specimen examined from Hokkaido [GP001] is from the northern limit of the distribution of the genus.

### Gamepenthes similis

Taxon classificationAnimalia ColeopteraElateridae

(Lewis, 1894)

C6E62BB3-3C9C-5544-8A82-8A83A4AAA07B

Figures 1C , 3E, F , 7C

Melanoxanthus similis Lewis, 1894: 182 (original description; type locality: Fukushima [Kisofukushima in Nagano Prefecture], Nikko in Tochigi Prefecture and Osaka in Osaka Prefecture, Honshu, Japan).
Gamepenthes similis (Lewis, 1894); Kishii 1959: 59 (changed generic status).

#### Note.

Ôhira (1970a) transferred *Melanoxanthus similis* to the genus *Gamepenthes*, although Kishii (1959) had already suggested that the species belongs to *Gamepenthes*.

#### Material examined.

1 male, Japan, Honshu, Fukushima Prefecture, 31 VII 1989, K. Shindô leg. [GS001]; 1 male, 2 females, Japan, Honshu, Kyoto Prefecture, Kyoto City, Sakyô Ward, Hirogawara-obana Town, Sasari-tôge Pass, 698 m, 35.27546°N, 135.72935°E, 1 VIII 2020, Kôichi Arimoto leg., by beating shurbs [male: GS008; females: GS009, GS010]; 1 male, Japan, Honshu, Nara Prefecture, Tenkawa Village, Tsubonouchi Forest Road, 21 VII 2008, Hisayuki Arimoto leg. [GS002]; 2 males, Japan, Honshu, Wakayama Prefecture, Tanabe City, Mt. Gomadan-zan, 18 VII 2019, Hisayuki Arimoto leg. [GS003, 004]; 1 male, 1 female, Japan, Honshu, Tottori Prefecture, Saihaku District, Daisen Town, Mt. Dai-sen, 30 VII 2016, Akihiko Watanabe leg. [male: GS005; female: GS006]; 1 female, Japan, Honshu, Hiroshima Prefecture, Hikimi-tôge Pass, 7 VIII 1983, T. Itô leg. [GS007].

#### Diagnosis.

Body robust (EI: 223–247, BI: 231–266). Prothorax black. Each elytron with yellow diagonal long marking basally. Antennomere II distinctly longer than wide; III longer than wide; IV robust (1.58–1.64 times longer than wide), shorter than II–III combined (0.67–0.76 times as long as II–III combined). Pronotum gradually widened posterad. Prosternal process weakly inclined dorsad (23–37°). Metacoxal plates not narrowed outwards, with outer edge wide. Apical edge of elytra rounded, without spines. Apex of parameres beyond apical-lateral hooks widely triangular (apex length 0.4 times width of parameres at expansion).

#### Measurements.

Male (6 spec.) BL: 4.22–4.65; BW: 1.21–1.31; MAE: 0.706–0.786; MBE: 0.487–0.576; OI: 136–145; PL: 1.11–1.25; PML: 0.924–1.07; PAW: 0.702–0.805; PW: 1.13–1.24; PI: 97.6–101; PWI: 154–165; EL: 2.84–3.21; EW: 1.21–1.31; EI: 234–247; BI: 246–266. Female (4 spec.). BL: 4.39–4.95; BW: 1.31–1.42; MAE: 0.728–0.819; MBE: 0.523–0.600; OI: 136–140; PL: 1.25–1.41; PML: 1.03–1.20; PAW: 0.736–0.832; PW: 1.20–1.37; PI: 103–105; PWI: 153–165; EL: 2.92–3.34; EW: 1.31–1.42; EI: 223–237; BI: 231–244.

#### Distribution.

Japan: Honshu, Shikoku, Kyushu.

### Gamepenthes versipellis

Taxon classificationAnimalia ColeopteraElateridae

(Lewis, 1894)

A28BC8D0-4419-540F-AA7B-A094F6006F21

Figures 1D , 2E , 3G , 3I , 4C, D , 7D

Megapenthes versipellis Lewis, 1894: 47 (original description; type locality: Chiûzenji in Tochigi Prefecture and Kashiwagi in Nara Prefecture, Honshu, and Junsai, Sapporo and Otaru in Hokkaido, Japan).
Gamepenthes versipellis (Lewis, 1894); Kishii 1958: 29 (changed generic status).
Megapenthes versipellis aberrant form nigerrimus Nakane, 1958: 86 (original description; type locality: Mt. Osore-zan, Aomori Prefecture, Honshu, Japan).
Megapenthes versipellis aberrant form sexpunctatus Nakane, 1958: 86 (original description; type locality: Mt. Osore-zan, Aomori Prefecture, Honshu, Japan).
Megapenthes versipellis aberrant form octopustularus Nakane, 1958: 87 (original description; type locality: Mt. Osore-zan, Aomori Prefecture, Honshu, Japan).
Megapenthes versipellis aberrant form interruptus Nakane, 1958: 87 (original description; type locality: Mt. Osore-zan, Aomori Prefecture, Honshu, Japan).
Megapenthes versipellis var. shirozui Kishii, 1958: 31 (original description; type locality: Mt. Kujyû-san, Ôita Prefecture, Kyushu, Japan).

#### Note.

The species exhibits elytral color variation. Based on this, Nakane (1958) and Kishii (1958) described four forms and one variant.

#### Material examined.

1 female, Japan, Hokkaido, Samani District, Samani Town, Mt. Apoi-dake, 9 VIII, 1974, H. Miyauchi leg. [GV001]; 1 male, Japan, Honshu, Nagano Prefecture, Mt. Kisokomaga-take, 31 VII 1976, Ryôji Toyoshima leg. [GV002]; 1 male, Japan, Honshu, Nagano Prefecture, Mt. Yatsuga-take, 26 VII 1979, Katsuhiko Kitagawa leg. [GV003]; 1 male, Japan, Honshu, Gifu Prefecture, Ôno District, Shôkawa Village, Ogamigô, 12 VIII 1995, N. Yuzawa leg. [GV004]; 1 male, Japan, Honshu, Nara Prefecture, Yoshino District, Kamikitayama Village, Mt. Ôdaigahara, 14 VII 1985, Hisayuki Arimoto leg. [GV005]; 1 male, 3 females, Japan, Honshu, Nara Prefecture, Yoshino District, Totsukawa Village, Asahi, Mt. Syakaga-take, 1450 m, 4 VIII 2019, Hisayuki Arimoto leg., from flowers of *Tilia japonica* [male: GV006; females: GV007–GV009]; 2 males, Japan, Honshu, Nara Prefecture, Kamikitayama Village, Amagase, 11 VII 2004, Hisayuki Arimoto leg. [GV010, GV011]; 1 male, Japan, Honshu, Nara Prefecture, Tenkawa Village, 23 VII 1989, H. Nomura leg. [GV012]; 1 female, Japan, Honshu, Hyôgo Prefecture, Shisô City, Haga Town, Tokura, Mt. Hyôno-sen, 1 VII 2015, S. Sugimoto leg. [GV013]; 1 female, Japan, Shikoku, Ehime Prefecture, Mt. Ishizuchi-san, 26 VII 1979, Kiyoshi Matsuda leg. [GV014]; 1 male, 2 females, Japan, Kyushu, Miyazaki Prefecture, Nishiusuki District, Gokase Town, Mt. Shiraiwa-yama, 1347 m, 32°34'13.7"N, 131°06'51.6"E, 30 VII 2011, Kôichi Arimoto leg. [male: GV015; females: GV016, GV017].

#### Diagnosis.

Body robust (EI: 226–243, BI: 206–222). Prothorax black in male; female with pronotum and hypomeron red, prosternum black. Each elytron with pale orange U-shaped marking basally; basal marking partly reduced in some. Antennomere II shorter than wide in male, slightly longer than wide in female; III shorter than wide in male, longer than wide in female; IV robust (1.48–1.72 times longer than wide in male, 1.66–1.75 times longer than wide in female), longer than II–III combined (1.72–1.99 times as long as II–III combined in male, 1.08–1.14 times as long as II–III combined in female). Prosternal process weakly inclined dorsad (17–26°). Metacoxal plates not narrowed outwards, with outer edge wide. Apical edge of elytra serrate. Apex of parameres beyond pre-apical expansions widely triangular (apex length 0.5–0.6 times width of parameres at expansion).

#### Measurements.

Male (8 spec.). BL: 6.60–7.53; BW: 1.92–2.19; MAE: 1.07–1.19; MBE: 0.709–0.831; OI: 141–154; PL: 1.98–2.33; PML: 1.73–1.96; PAW: 1.10–1.22; PW: 1.92–2.19; PI: 103–110; PWI: 172–184 ; EL: 4.30–5.03; EW: 1.88–2.11; EI: 229–243; BI: 206–222. Female (9 spec.). BL: 7.08–9.24; BW: 2.03–2.60; MAE: 1.12–1.30; MBE: 0.753–0.907; OI: 142–149; PL: 2.20–2.77; PML: 1.89–2.38; PAW: 1.15–1.39; PW: 2.03–2.60; PI: 106–112; PWI: 176–191; EL: 4.64–6.04; EW: 2.02–2.58; EI: 226–241; BI: 207–219.

#### Distribution.

Japan: Hokkaido, Honshu, Shikoku, Kyushu. Russia: Kunashir Island. This species was also recorded from China and Oriental region (Cate 2007). Schimmel (2003, 2004, 2006) and Schimmel and Tarnawski (2009) recognized 18 species from China and Indochina: however, *G. versipellis* had not been confirmed from the area. Therefore, the records of *G. versipellis* from China and the Oriental region were probably based on misidentifications of allied species, such as *G. hubeiensis* Schimmel, 2003, *G. holzschuhi* Schimmel, 2003, *G. kresli* Schimmel & Tawnawski, 2009, *G. sausai* Schimmel, 2003 and *G. sichuanensis* Schimmel, 2003.

### Gamepenthes yoshidai

Taxon classificationAnimalia ColeopteraElateridae

Ôhira, 1995

534FFB0F-D51F-5EFE-AEEC-09F9A2703259

Figures 1E , 4F , 7E

Gamepenthes yoshidai Ôhira, 1995b: 27 (original description; type locality: Mt. Tsurugi-san, Tokushima Prefecture, Shikoku, Japan).

#### Material examined.

***Paratype*.** 1 female, Japan, Shikoku, Tokushima Prefecture, Miyoshi City, Mt. Tsurugi-san, Minokoshi, 31 VII 1982, Yûji Kurota leg.

#### Non-type material.

2 males, Japan, Shikoku, Tokushima Prefecture, Naka District, Naka Town (formerly Kisawa Village), Mt. Takashiro-yama, 31 VII 1993, Mitsuo Kakô leg. [GY001, GY002].

#### Diagnosis.

Body slender (EI: 259–265, BI: 254–282). Prothorax black; hind angles of pronotum yellow. Each elytron with three separate yellow markings basally. Antennomere II shorter than wide in male, almost as long as wide in female; III shorter than wide in male, longer than wide in female; IV elongate (1.92–2.06 times longer than wide in male, 2.17 times longer than wide in female), longer than II–III combined (1.27–1.77 times as long as II–III combined). Prosternal process strongly inclined dorsad (43–50°). Metacoxal plates narrowed outwards on lateral half, with outer edge narrow. Apical edge of elytra rounded, without spines. Apex of parameres beyond pre-apical expansions widely triangular (apex length 0.3–0.4 times width of parameres at expansion).

#### Measurements.

Male (2 spec.). BL: 6.96–7.29; BW: 1..87–1.98; MAE: 1.17–1.21; MBE: 0.711–0.752; OI: 161–164; PL: 1.72–1.87; PML: 1.51–1.65; PAW: 1.17–1.23; PW: 1.74–1.87; PI: 98.8–100; PWI: 148–152; EL: 4.85–5.13; EW: 1.87–1.98; EI: 259; BI: 275–282. Female (1 spec.). BL: 7.20; BW: 1.89; MAE: 1.15; MBE: 0.743; OI: 154; PL: 1.98; PML: 1.71; PAW: 1.16; PW: 1.82; PI: 109; PWI: 157; EL: 5.02; EW: 1.89; EI: 265; BI: 254.

#### Distribution.

Japan: Shikoku. This species may be endemic to Shikoku, Japan.

## Discussion

The holotype of *N. alpicola* has an oval head capsule in lateral view (Fig. 5E), inferior mouth-parts (Fig. 5E), mesocoxal cavity open to the mesepimeron and mesepisternum (Fig. 5F), mesosternum separated by suture from metasternum (Fig. 5F), and claws simple and without basal setae (Fig. 5I). These features are diagnostic for members of the subfamily Elaterinae (Stibick 1979). Moreover, the holotype has the following key features for the genus *Gamepenthes* of the tribe Megapenthini (Ôhira 1970a; Schimmel 2003): supra-antennal carina compete and depressed medially (Fig. 5A), vertical distance between supra-antennal carina and labrum narrowed medially in anterior view (Fig. 5A), antennomeres II and III short (Fig. 5B), antennomeres IV–X serrate (Fig. 5B), hind angles of pronotum unicarinate (Fig. 5C), procoxal cavity partly closed by a projection of the hypomeron, prosternal process concave between procoxae (Fig. 5D) and with subapical tooth (Fig. 5E), and elytral surface with small tubercle-like prominences on anterior edge of punctures (Fig. 2C) (the elytral surface clearly granulated in Kishii 1966). Kishii (1987) stated that tarsomere IV of *N. alpicola* was slightly enlarged apically, but the tarsomere IV of the holotype is simple, which matches the diagnosis of *Gamepenthes* and Megapenthini (Fig. 5I). Therefore, we concluded that *Nipponodrasterius* should be a junior synonym of *Gamepenthes*.

We found a slide of the male genitalia labeled as belonging to the holotype of *N. alpicola* (matching specimen number and label information) in OMNH (Fig. 5K, L), although the sex and genitalia of the holotype were not mentioned in the original description. We found that *N. alpicola* should be a junior synonym of *G. pictipennis* by comparing the aedeagi of the Japanese *Gamepenthes* species (Figs 5J, 6, 7) and their aedeagus shown in Ôhira (1970b, 1995a). *Gamepenthes pictipennis* is also distinguished from its congeners by a combination of body size, antennae, prosternal process, metacoxal plates, and apical edge of the elytra (see above key and diagnoses). The holotype of *N. alpicola* shares these features (Fig. 5). We also found that *G. pictipennis* has variable pronotum and elytral coloration, ranging from a black base with clear yellow markings (Figs 1B, 2D) to having fewer yellow markings (Fig. 2A) to entirely black (Fig. 2B). Even the black specimens show slight yellow markings, and the hind angles of the pronotum and anterior parts of that elytra are slightly yellow tinged (Fig. 2B), as in the holotype (Figs 2C, 5C). We conclude the holotype of *N. alpicola* is a melanic specimen of *G. pictipennis*.

## Supplementary Material

XML Treatment for Gamepenthes

XML Treatment for Gamepenthes ornatus

XML Treatment for Gamepenthes pictipennis

XML Treatment for Gamepenthes similis

XML Treatment for Gamepenthes versipellis

XML Treatment for Gamepenthes yoshidai
